# Triploidy in a pair of hybridizing salamanders at the far end of the speciation continuum

**DOI:** 10.1093/jhered/esag006

**Published:** 2026-01-19

**Authors:** Jan W Arntzen, Evan McCartney-Melstad, Spartak N Litvinchuk, Ben Wielstra

**Affiliations:** Institute of Biology, Leiden University, 2333 BE Leiden, The Netherlands; Naturalis Biodiversity Center, 2333 CR Leiden, The Netherlands; Nutcracker Therapeutics Inc., Emeryville, CA, United States; Institute of Cytology of the Russian Academy of Sciences, St. Peterburg, Russia; Institute of Biology, Leiden University, 2333 BE Leiden, The Netherlands; Naturalis Biodiversity Center, 2333 CR Leiden, The Netherlands

**Keywords:** backcross hybrids, erythrocyte morphometrics, gene capture, metric multidimensional scaling, single nucleotide polymorphisms, *Triturus cristatus* x *T. marmoratus* hybrids

## Abstract

Ecological and genetic interactions of species at the far end of the speciation continuum can often be studied in sympatry. *Triturus cristatus* (C) and *Triturus marmoratus* (M) are two deeply differentiated yet hybridizing salamander species that engage in a mosaic distribution over a wide zone of range overlap in the west of France. Interspecies hybrids are easy to distinguish from both parentals and occur at ca. 3% of the total breeding population. Most hybrids are thought to be F_1_ diploids (CM). We aim to identify triploid F_1_ hybrids with single nucleotide polymorphism (SNP) data and determine their frequency. Special attention to the genus *Triturus* is warranted because triploidy might in principle constitute an escape route to the enigmatic chromosome-1 syndrome that constitutes a long-term 50% genetic load. Species-specific signals for a panel of 30 SNPs, analyzed with metric multidimensional scaling, suggest that triploid frequency is one out of 26 (3.8%), although a frequency of 18% was found in a larger sampling (*n* = 100) that paid particular attention to aberrant hybrid phenotypes. Triploids with a CMM genetic configuration appear more frequent than the CCM counterpart. The triploid status could be confirmed for two CMM females, either by target capture or cellular morphometric data. One of these two triploid individuals relocated in the field showed a hybrid phenotype leaning towards *T. marmoratus*, an exceptional life span (17+ yrs), length (187 mm) and the frequent skipping of annual breeding opportunities. Future research on the *T. cristatus*—*T. marmoratus* system should consider the different classes of interspecies hybrids.

## Introduction

The speciation process involves the gradual build-up of genetic, ecological, and behavioural incompatibilities and increasing reproductive isolation (Coyne and Orr 2004). Genetic divergence is a by-product of genetic isolation, which is typically accomplished by vicariance and divergent evolution (Mayr 1963). When, after a period of genetic isolation, gene pools regain contact, any evolved reproductive barrier is put to the test. The outcome of this natural experiment may range from a full merger to the complete genetic isolation of the two gene pools (Wu and Ting 2004; Stankowski and Ravinet 2021).

Incompletely isolated species enable investigation of the genetic mechanisms and evolutionary forces that maintain their identity in the face of ongoing gene flow. Systems positioned towards the far end of the speciation continuum, with deeply diverged yet hybridizing species, provide opportunities to study the final stages of the speciation process (Barton 2020). A regular observation for related species that hybridize upon secondary contact is that they geographically exclude one another, either with a narrow clinal hybrid zone forming their abutting range borders, or in a mosaic hybrid zone in which taxa map onto patches of interdigitated habitat (Barton 1979; Harrison 1986; Jiggins and Mallet 2000). When, however, species have diverged ecologically to the extent that interspecific competition is reduced, sympatry may prevail over parapatry and hybrid individuals, whilst rare, may be found scattered across the area of range overlap (Key 1981; Barton and Hewitt 1985, see also Schluter et al. 2025 and references therein).

**Fig. 1:**
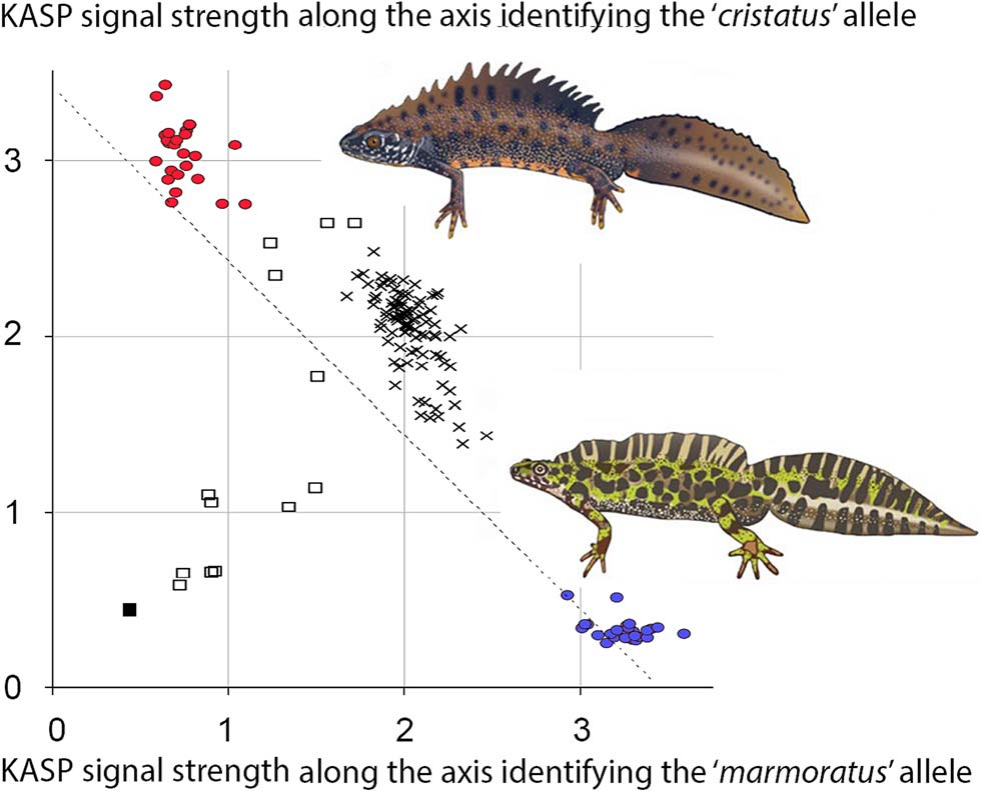
Bivariate plot of KASP-scores (in fluorescence units) for the locus *abl*, the first out of 30 markers in the first batch of analyzed individuals. Individuals top left are classified as homozygous for the *abl*^C^ allele that is typical for *Triturus cristatus* (CC, round symbols in red, upper animal drawing), individuals bottom right are homozygous for the *abl*^M^ allele that is typical for *T. marmoratus* (MM, round symbols in blue, lower animal drawing) and those in the centre are classified as heterozygous (CM, crossed symbols). The solid square symbol denotes the position of a blank sample. A signal strength threshold value of 3.4 in city-block distance is applied as illustrated by the interrupted line. Open square symbols denote 13 individuals for which no genotype call was made, presumably either on account of a strong but intermediate signal (four data points in between the CM and MM clusters) or signal strength below the threshold. It is here considered that the former group might represent F_1_ hybrids with a duplicated *abl*^C^ allele (CCM), as might arise from e.g., gene duplication, trisomy, or triploidy. The animal drawings are by Bas Blankevoort, Naturalis Biodiversity Center.

The ‘large-bodied newts’ *Triturus cristatus* (northern crested newt, ‘C’) and *T. marmoratus* (marbled newt, ‘M’) are phenotypically very different salamander species (see Fig. 1) at the far end of the speciation continuum (Arntzen et al. 2021), that engage in a wide zone of range overlap and hybridization in the west of France (Lescure and de Massary 2012; Arntzen 2023a, 2023b). The species interaction is characterized by ecological and behavioural differentiation and limited gene flow beyond the F_1_ hybrid generation. Hybrids occur at a frequency of ca. 3% of the total breeding population and are easy to recognize by eye, as are both parentals (Vallée 1959). Backcrossing and introgression are rare (Arntzen and Wallis 1991; Arntzen et al. 2021).

Hybridization accompanied by genome duplication is a powerful evolutionary driver that has significantly influenced vertebrate divergence (Yu et al. 2024). However, its mechanisms and the evolutionary success of polyploids in animals remain poorly understood. Amphibians are the only vertebrate group with bisexual polyploid species coexisting with diploid ancestral forms, rendering them unique models for studying genomic instability in the context of speciation (Dufresnes et al. 2019). A key challenge is to understand the molecular-genetic determinants that allow newly formed allopolyploid lineages to overcome hybrid sterility and establish themselves in populations where they have to compete with diploid ancestral forms. Typically, during the early stages of reticulated (e.g., hybridogenous) speciation in amphibians, triploids occur occasionally and these triploids can be fertile (Litvinchuk et al. 2016). Cases of natural spontaneous triploidy are known for the genus *Triturus* and close relatives, but these are usually associated with autopolyploidy rather than allopolyploidy (Litvinchuk et al. 1998, 2001), whereas in laboratory crossbreeding triploids are relatively common (Lantz and Callan 1954; Mancino et al. 1976). The main problem with such studies is, however, that researchers studying natural hybridization tend to ignore possible ploidy changes.

We here address the questions if some of the hybrids in the *T. cristatus—T. marmoratus* system may be triploid, how common these triploids are and how to distinguish them from diploid F_1_ and backcross hybrids. To this aim we use species-diagnostic SNP (single nucleotide polymorphism) data gathered with Kompetitive Allele-Specific PCR (KASP) genotyping. Whilst this methodology is optimized for analyzing diploid genotypes, we employ a multivariate method to infer triploid genetic configurations. We also use the independent techniques of target capture and erythrocyte morphometrics to confirm the existence of triploid hybrids identified with KASP genotyping. Finally, we describe morphological and behavioural characteristics of a female triploid hybrid that was relocated at its breeding pond.

## Materials and methods

The area of research is the ‘département’ (dept.) Mayenne in the west of France, where *T. cristatus* and *T. marmoratus* share breeding sites and infrequently hybridize. The identification of hybrids was based on their ventral colouration characteristics that are intermediate to that of the parental species (see the extensive photographic documentation in Arntzen et al. 2021). Tail tip tissue samples for molecular genetic analyses were gathered starting in 1980, with special attention to aberrant phenotypes that were suspected to be (rare) F_2_-backcross hybrids. Sample duplication was minimized by spreading the work over time and space and by using individual electronic tags (Passive Integrated Transponders, or PIT-tags) in population studies (Arntzen 2025b). Data on individual ploidy level were gathered with three methods. First, KASP genotypes were obtained in three batches for 30 nuclear genetic SNP markers positioned in 3′-untranslated regions of protein-coding genes (taken form Arntzen et al. 2021) for altogether 33 *T. cristatus*, 67 *T. marmoratus* and 143 hybrids. Material for batch 1 was collected before the year 2000 whereas other samplings (i.e., batches 2 and 3) were more recent (Table 1). The other techniques used were target capture and cellular morphometrics. KASP involves fluorescence-based genotyping of individual SNPs in uniplex assays (Semagn et al. 2014). Next to a common reverse primer, assays include two allele-specific forward primers with a final base complementary to one of the two potential SNP variants. The allele-specific primers possess unique tail sequences and two distinctly fluorescently labelled sequences present in the KASP master mix are complementary to each tail sequence. These labels are originally quenched and get activated when incorporated during PCR cycles, with further cycling causing signal intensity to increase.

**Table 1:** Overview of sampling on *Triturus cristatus, T. marmoratus,* and phenotypic interspecies hybrids in dept. Mayenne, France used for KASP-genotyping. Samples not considered due to low signal strength are listed after the plus-sign. For KASP batches 1 and 2 see also Arntzen et al. (2021).

Sampling	Phenotypic classification				Failed (%)	Triploids
Batch	Period	*T. cristatus*	*T. marmoratus*	Hybrid		
1^a^	1980–1999	18 + 1	23	100 + 16	10.8	18
2	2000–2009	7	38	6 + 1	1.9	None
3	≥2010	7	6	20	0	1

^a^Sampling with particular attention to aberrant intermediate phenotypes.

KASP genotyping has been optimized for identifying diploid genotypes, i.e., homozygotes (say CC and MM) and heterozygotes (CM), whereas triploid complements (CCM and CMM, if these were to exist) are not normally considered. Data points ‘uncalled’ by the KASP software Kraken (LGC Biosearch Technologies) may reflect a substandard signal due to low quality DNA, but could also represent an adequate but difficult-to-classify signal, on account of a position in between clouds of homozygous and heterozygous genotypes. For a single locus example see Fig. 1. Although such ambiguous signals may be genuine outliers in the clouds of points for the homozygous or the heterozygous condition, they could also represent triploid individuals.

We searched for a consistent intermediate signal across markers, in line with expectations for diploid F_1_ hybrids and backcrosses versus triploid F_1_ hybrids. The analysis was based upon the KASP signal and ignored the genotype classification provided by the associated software (Semagn et al. 2014). An empirically supported threshold for KASP signal strength of 3.4 was applied as determined for batches 1 and 2 (Arntzen 2024). Results for 18 individuals with signals averaging below the threshold were considered failed. The remaining KASP-scores were subjected to metric multi-dimensional scaling (mMDS) with Primer v7 software following the manual (Clarke and Gorley 2015). Briefly, a ‘resemblance matrix’ of Euclidian distances was constructed from normalized data for each of three data analytical batches and mMDS was run with 500 random starts and a minimum stress value of 0.001. To allow comparison of results across batches, the scores over the first axis were scaled with the average value for phenotypically identified *T. cristatus* and *T. marmoratus* set at zero and unity, respectively.

We also carried out a simple simulation to gain an impression of where various hybrid classes might be expected to show up along the first mMDS axis, on the assumption that the results scale linearly. Scores for reference *T. cristatus* and *T. marmoratus* were randomly combined as simulated parentals (CC and MM), F_1_ hybrids (CM), F_1_ triploid hybrids of either kind (CCM and CMM) and backcross hybrids (‘B’) towards *T. cristatus* (BC) and *T. marmoratus* (BM). Normal distributions were fitted to 1000 combinations per class, for comparison with the genetic profile observed from the original data. It was also attempted to relocate phenotypic hybrid individuals for which the KASP data suggested that they were triploids. A single such individual was found, as recognized by its PIT-tag.

Second, we employ the NewtCap target capture protocol (De Visser et al. 2025a) to obtain sequence data of exons for ca. 7000 nuclear DNA markers (details in Wielstra et al. 2019) for ten hybrids that were also genotyped by KASP (individuals F_1_A-F_1_J in Supplementary Information 1). In brief, this encompassed shearing ca. 10 000 ng of DNA per sample to c. 200–500 bp on a BioRuptor NGS (Diagenode) and dual-end size selection (0.8–1.0X) with Solid Phase Reversible Immobilization beads. This was followed by dual-indexed library preparation from 375 to 2000 ng of size selected DNA using the KAPA LTP library prep kit (Bayona-Vásquez et al. 2019). We then performed target enrichment on the pooled library using a custom probe set based on *Triturus* transcriptome data with the MyBaits v4.0 kit, Arbor Biosciences Ref# 170210–32. The enriched library was 150 bp paired-end sequenced on the NovaSeq 6000 platform (Illumina Inc., San Diego, CA, USA).

We visualized ploidy in these data by investigating the frequency of sequence reads covering both alleles at heterozygous sites. For a typical biallelic diploid heterozygous site, each allele should be covered roughly equally by sequencing reads, whereas for a triploid individual the less commonly sequenced allele should be present at ~33%. Sequence reads were trimmed for adapter contamination and quality using Trimmomatic v0.36 (Bolger et al. 2014) and mapped to a *T. carnifex* reference assembly (Wielstra et al. 2019) with BWA-MEM v.7.15-r1140 (Li 2013). Sample gVCFs were generated using HaplotypeCaller and joint genotyping was performed using GenotypeGVCFs from GATK v3.7 (Poplin et al. 2017). The raw VCF was filtered for biallelic SNPs passing the following quality filters: QD < 2.0, MQ < 40.0, FS > 60.0, MQRankSum < −12.5, ReadPosRankSum < −8.0, and QUAL <100. Heterozygous genotypes with GQ >30 and depth >20 were analyzed for allelic representation by processing the genotype AD fields with a custom python script.

Thirdly, we prepared blood smears for 49 adult newts in series 1 (27 hybrids along with seven *T. cristatus* and four *T. marmoratus*) and series 2 (11 hybrids). The microscopy slides were stained with Diff-Quik stain to facilitate the measurement of erythrocytes’ morphometric characteristics. The cells were measured under a Axioscop-DFS360 microscope with a digital camera using ImageJ software available at https://imagej.nih.gov/ij. Thirty erythrocytes per individual were randomly chosen for the measurement (in μm) of length (El), width (Ew), nuclear length (Nl), and nuclear width (Nw). Planimetric erythrocytes size (Es) and nuclei size (Ns) were computed as Es = El × Ew × π/4 and Ns = Nl × Nw × π/4. Measurements were averaged per individual. One field-relocated hybrid suspected to be triploid (see above) was studied in duplicate, although this was not known to the investigator SNL. This individual’s body sizes over time (SVl, snout-vent length up to and including the insertion of the hind legs, in mm) were used to construct a Von Bertalanffy growth curve with the R programme nls.multstart (Padfield et al. 2018) and compared with data from the literature (Cogălniceanu et al. 2020). Body condition (BCo) is defined as the residual of the linear regression of log mass (M, in g) on log SVl (in mm) with *T. cristatus* females as the reference (BCo = logM −2.827*logSVl −4.198, *n* = 465) with all data obtained from the pond in which a hypothesized triploid hybrid female was found.

## Results

**Fig. 2:**
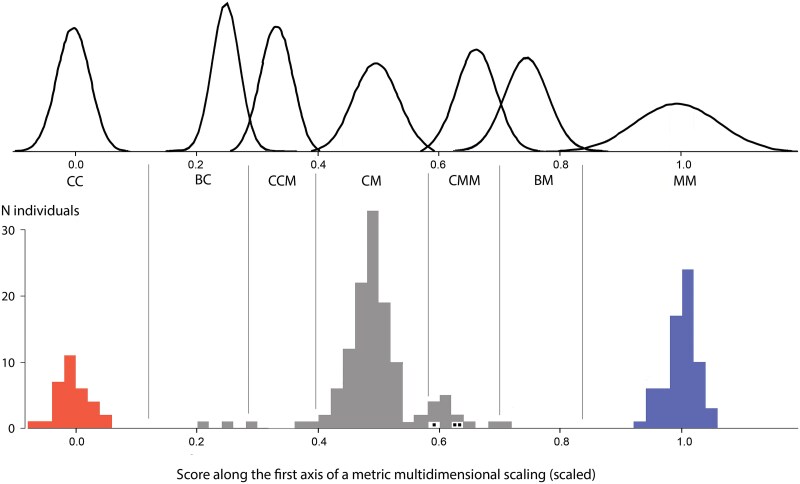
Histogram of scores along the first axis by metric multidimensional scaling (mMDS) of KASP data on 30 species diagnostic nuclear loci for 225 *Triturus* newts. Field-recorded phenotypes are *Triturus cristatus* shown by red bars, *T. marmoratus* shown by blue bars and interspecific hybrids shown by grey bars. Hybrid individuals inferred to be triploids are shown in white with one black dot for identification with erythrocyte morphometric data and with two black dots for identification by target capture (details see text). Values are scaled from zero for the average of phenotypic *T. cristatus* to unity for phenotypic *T. marmoratus*. The curved lines in the upper panel show the normal distributions for simulated offspring as *T. cristatus* (CC), *T. marmoratus* (MM), diploid (CM), triploid interspecific F_1_ hybrids (either CCM or CMM) and backcross hybrids (either BC or BM). Thin vertical lines indicate mMDS first axis values at which separation amongst genotype clusters is most straightforward.

The histogram for scores over the first mMDS axis is distinctly trimodal, with peaks corresponding to *T. cristatus*, hybrids and *T. marmoratus*, with no phenotype–genotype discrepancies (Fig. 2). The number of individuals with a KASP signal strength averaging below the 3.4 threshold (Fig. 1) was high in batch 1 (17 out of 158, 10.8%) and low in the combined batches 2 and 3 (one out of 85, 1.2%). In batch 1 the scores along the second mMDS axis strongly correlate with KASP signal strength (r = −0.827, *P* < 0.0001). The unprocessed KASP-scores are presented in Supplementary Information 1, along with species identity, sex, pond of origin, and mtDNA configuration (in addition to Arntzen et al. 2021).

Most phenotypic hybrids are best interpreted as diploid F_1_’s, as supported by the simulated frequency distributions. Yet, 19 phenotypic hybrids fitted the distribution for simulated triploid hybrids and backcrosses better than that for diploid hybrids. Discrimination between triploid and backcross genotypes is not absolute, but the most straightforward classification yields two BC and one BM backcrosses and three CCM and 13 CMM triploids (Fig. 2). The one hybrid eventually relocated in the field was classified as CMM. The classification as BC or CCM versus BM or CMM was significantly different from expected equal frequencies (*n* = 5 versus *n* = 14, binomial test, z = 1.835, *P* < 0.05).

**Fig. 3:**
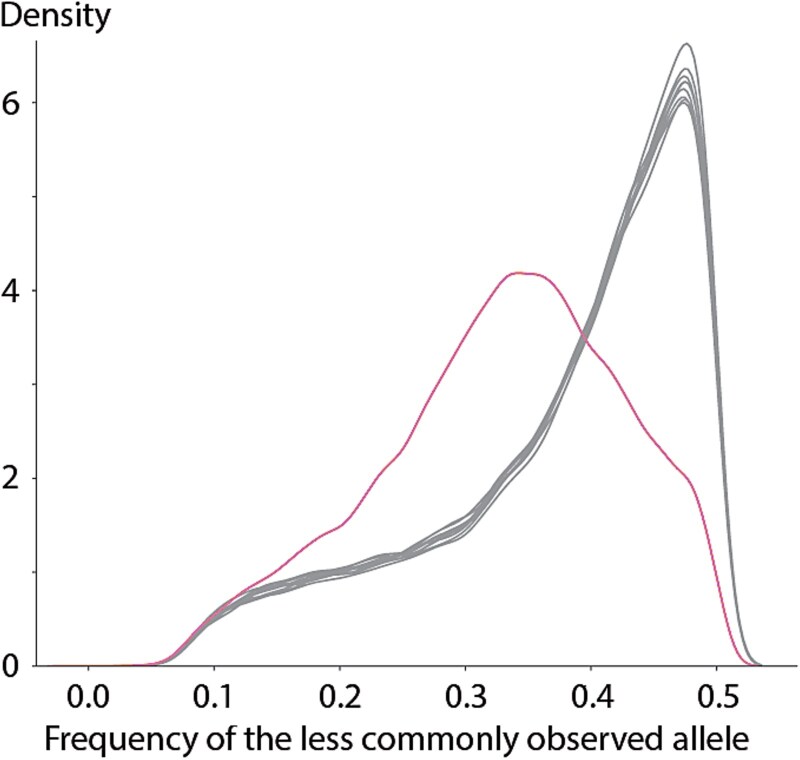
Target capture results for ten *Triturus cristatus x T. marmoratus* hybrids. Nine individuals (in grey) show a less commonly observed minor allele frequency distribution (expressed as a probability density function) with a peak close to 50%, in line with diploidy, whereas the tenth individual (in purple) shows a peak close to 33%, in line with triploidy.

Amongst phenotypic hybrids, significantly more triploids and backcrosses were inferred in batch 1 than in the batches 2 and 3 (18 out of 100 versus one out of 26, G-test for independence, G = 7.676, *P* < 0.01). Out of ten F_1_ hybrids for which target capture data were produced, nine showed a less commonly observed minor allele frequency distribution with a peak close to 50%, in line with diploidy. The tenth individual shows a peak close to 33%, in line with triploidy (Fig. 3). The field note for this female that carried marmoratus type mtDNA (Supplementary information 2) was ‘Possibly a backcross hybrid; black belly with a yellow shine in the centre’.

**Fig. 4:**
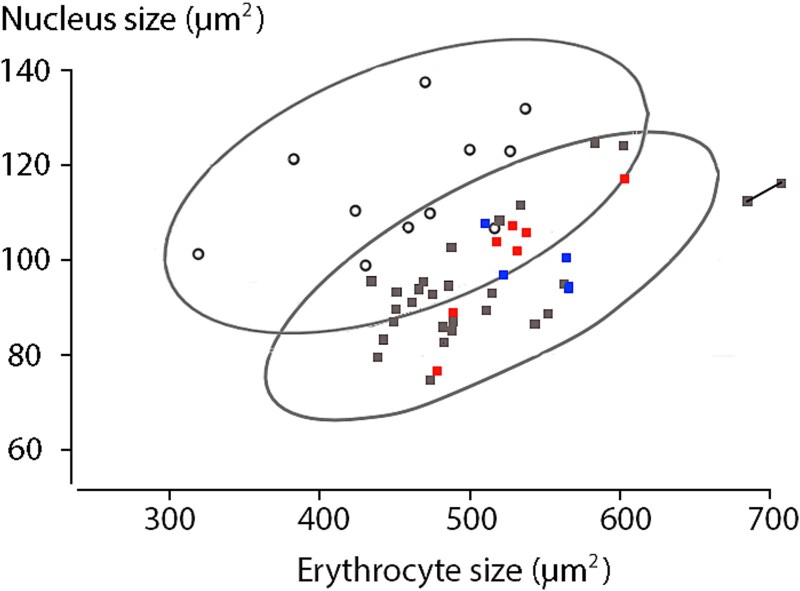
Bivariate plots of erythrocyte size (horizontal axis) and nucleus size (vertical axis) observed for 49 *Triturus* newts from dept. Mayenne, France*.* The ellipses describe the 95% confidence interval of the mean, separately for series 1 (solid square symbols) and series 2 (open round symbols). *Triturus cristatus* are shown in red, *T. marmoratus* in blue and interspecies hybrids in grey. Note that measurements for one outlier individual were done in duplicate. This individual, inferred to be a triploid F_1_ hybrid, is shown by habitus in Fig. 5.

Erythrocyte morphometric data were obtained in two series for which results are analyzed separately because statistically significant differences (outlier excluded) were found for nuclear size (t-test, t = 4.829, df = 46, *P* < 0.0001) and erythrocyte size (t = 2.531, *P* < 0.05) between series. The 95% confidence intervals of the mean encompass all individuals except for the outlier (Fig. 4). This individual was studied in duplicate with near-equivalent results. For data details see Supplementary Information 3.

**Fig. 5:**
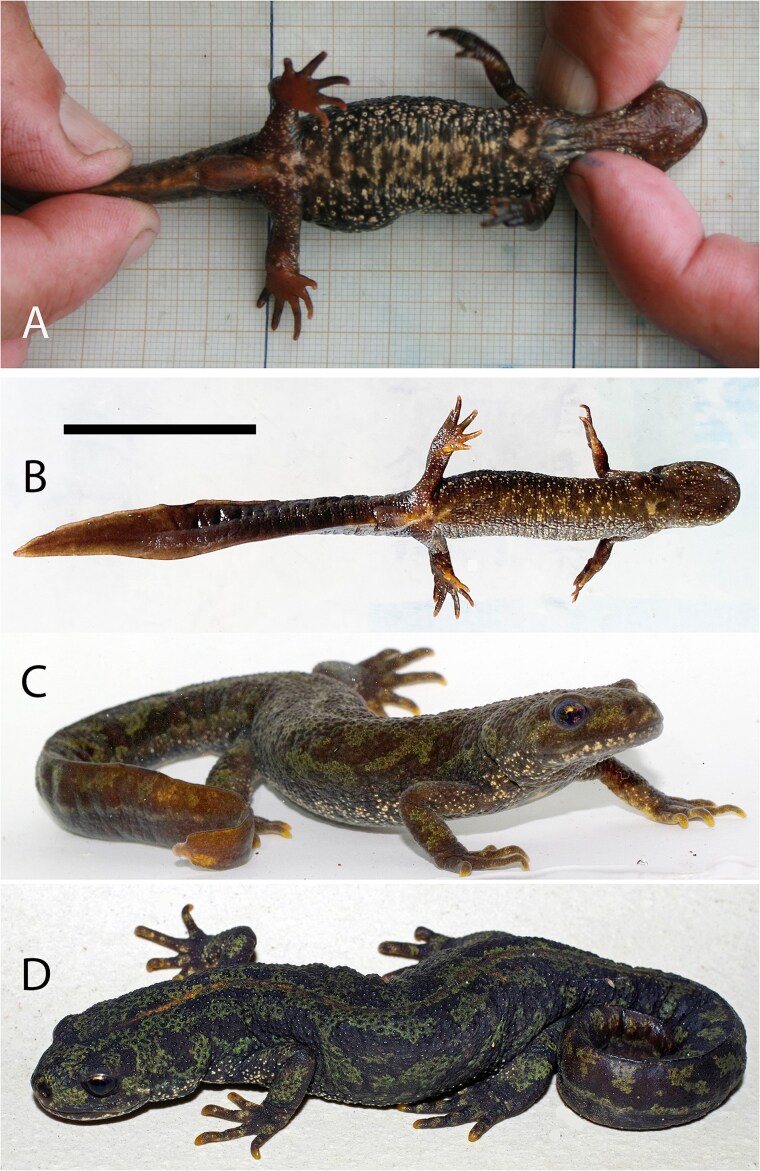
A female *Triturus cristatus* x *T. marmoratus* F_1_ hybrid inferred to be a triploid by the multivariate analysis of KASP genetic data (Fig. 2) and by the size of its red cells and nuclei (Fig. 3). The individual is shown for when it was first encountered in spring 2013 at the age of seven years (A), in spring 2023 at age 17 (B), and in the terrestrial phase in the same year (C and D). The black bar represents five cm. The malformation at the tail tip (best visible in C) stems from the clipping of the tail for preparing a blood smear followed by regeneration. Photo credit for C and D: Sergé Bogaerts.

**Fig. 6:**
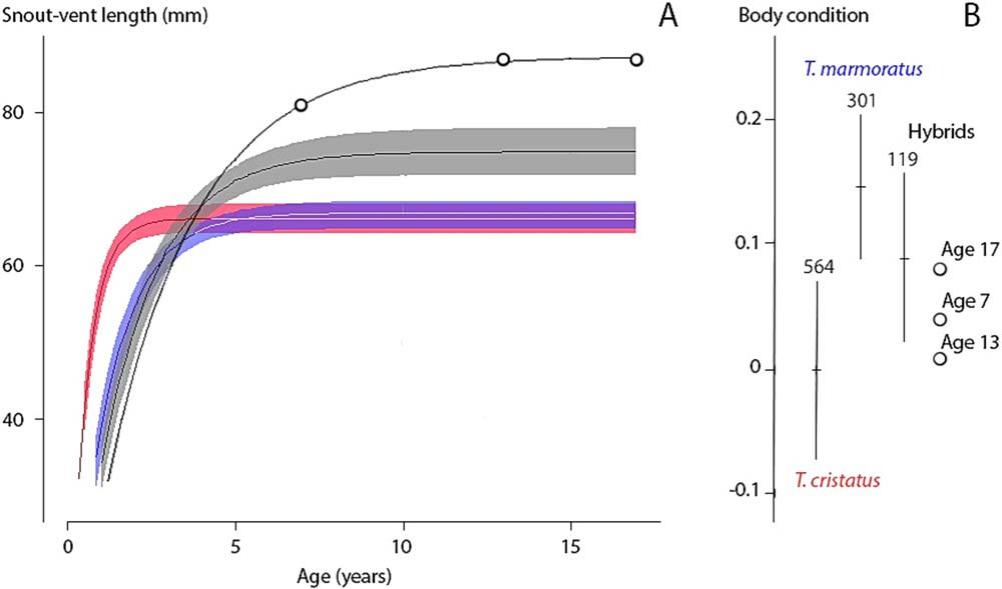
Quantitative assessment of size, growth, and body condition for large bodied newts from the French department Mayenne. Panel A —Von Bertalanffy growth curves with 95% confidence interval for *Triturus cristatus* in red, *T. marmoratus* in blue and interspecific hybrids in grey, recalculated from published skeletochronology data (Francillon-Vieillot et al., 1990; Cogălniceanu et al. 2020). The age of a hybrid identified as triploid (Fig. 5) was seven years in 2013, 13 years in 2019 and 17 years in 2023 and has an individual growth curve fitted (single thin black line with open round symbols). Note that this individual was not encountered in another ten study years despite intensive samplings with high annual detection probabilities (details see text). Panel B—Body condition of *Triturus* females in the pond where the triploid female of Fig. 5 was also found. Shown are averages ± one standard deviation, with sample sizes on top of the bars; for definition see text. Whilst the body condition of hybrids is intermediate to that of both parental species, that of the documented triploid hybrid (open round symbols) is comparable to other, mostly diploid hybrid individuals.

The above individual’s hybrid phenotype was apparent from mixed colouration characteristics and was upon first capture in 2013 field-classified as a possible backcross hybrid in the direction of *T. marmoratus*. It had a distinct, green dorsal marbled pattern as in *T. marmoratus*, whereas *T. cristatus* is uniformly dark. Its ventral colouration was dark with white stipples as in *T. marmoratus*, yet with a little yellow or orange as typical for *T. cristatus* shining through (Fig. 5). When first caught this individual was seven years old, as was established by skeletochronology analysis (Cogălniceanu et al. 2020) with a SVl of 81 mm. Six years later SVl was 87 mm and ten years on SVl was 87 mm with a total length of 187 mm. Body lengths measured including the cloaca (for comparison with other studies) were 89 mm, 95 mm and 96 mm, respectively. Body mass went up from 17.4 g in 2013 to 19.7 g in 2019 and 23.2 g in 2023. Sizes of this individual compared against generalized growth curves reconstructed for *T. cristatus, T. marmoratus,* and hybrids from the same study area show a deviation towards large size and continued growth (Fig. 6). Body condition for the *T. cristatus* and *T. marmoratus* was different, with the triploid individual and other hybrids taking an intermediate position.

## Discussion

According to a standing hypothesis, whole-genome duplications provided a substrate for the increased complexity of metabolic pathways (Qi et al. 2024; Yu et al. 2024) as has been particularly well documented for glycolysis (Steinke et al. 2006), but the consequences of polyploidization may not always be obvious. Molecular studies often reveal significant shifts in the expression of genes, which can lead to differences in physiology, ecology, behaviour, and morphology (Steinke et al. 2006; Dufresnes et al. 2019; Cadart et al. 2023; Yu et al. 2024). For example, genome duplication in the allotetraploid frog *Xenopus laevis* causes non-additive changes in the expression and interactions of several pluripotency factors to form a newly integrated regulatory network (Phelps et al. 2023). Moreover, the evolution of polyploid genomes is characterized by subgenomic partitioning, neo- and sub-functionalization of duplicated genes (Lv et al. 2025), as well as maternal dominance of expression and epigenetic silencing (Xu et al. 2023), which were not observed in diploid ancestors. Ploidy shifts have also been shown to directly correlate with changes in reproductive modes (Lu et al. 2022), which may lead to the isolation of genomes and subsequent independent evolution, i.e., speciation (Verta et al. 2021; Bartolić et al. 2024). The dynamic evolution of satellite DNA and nucleolar organizers has been found to reflect increased genomic plasticity after duplication (Fornaini et al., 2023). Polyploidy can accelerate diversification where recurrent autopolyploidization facilitated radiation exposure under extreme conditions, as in Tibetan fish (Zhan et al. 2014; Li and Guo 2020; Li et al. 2024). Polyploidy may enhance stress tolerance (Spoelhof et al. 2020; Van de Peer et al. 2021) and it has been noted that the genomic and phenotypic buffering provided by polyploidy increases resistance to abiotic and biotic stressors, which may give polyploids an advantage under rapid environmental changes (Van de Peer et al. 2021).

In addition to polyploidy, genetic introgression resulting from hybridization plays a significant role in adaptation (Shaw 2002; Chan and Levin 2005; Currat et al. 2008; Plötner et al. 2008; Excoffier et al. 2009; Toews and Brelsford 2012; Zieliński et al. 2013; Barley and Cole 2025). For example, recent studies have shown that adaptive introgression plays an important role in convergent adaptation to hypoxia in Tibetan fishes by ensuring the transfer of alleles of HIF-signalling pathway genes (Storz and Signore 2021; Qian et al. 2023). Hybridization alters the fitness landscape by facilitating access to new adaptive peaks (Patton et al. 2022), whilst co-expression networks and allele-specific expression alter the depth of reorganization of regulatory landscapes (Runemark et al. 2025).

Amongst amphibians, polyploidy is quite common in natural populations. For example, triploid individuals can be found within populations of diploid species. Spontaneous autopolyploidy has been found in 23 species from 15 genera and nine families (Eto et al. 2016; Litvinchuk et al. 2016, 2017; Hernández-Guzmán and Arias-Rodriguez 2025). Recently, the first autopolyploid tetraploid lineage was discovered in the genus *Microhyla* (Chen et al. 2025). Triploid individuals have been found in hybrid zones of diploid species of the genera *Lithobates* and *Anaxyrus* (Feder 1979; Green and Delisle 1985). Furthermore, polyploid (2-5n) individuals are common amongst diploid-polyploid hybridogenetic forms in the genera *Ambystoma* and *Pelophylax*, characterized by various types of clonal reproduction. Finally, amongst anurans, 53 bisexual allopolyploid species (3n, 4n, 8n, and 12n) are known, belonging to 15 genera and ten families. Polyploid individuals may also arise from hybridization between diploid and polyploid species (Litvinchuk et al. 2016).

We recently used diagnostic nuclear genetic markers to study hybridization and introgression in the deeply differentiated salamander species *T. cristatus* and *T. marmoratus* with large samples studied for few loci and smaller samples studied for many loci (Arntzen et al. 2021; Arntzen 2024). This confirmed expectations from morphology and molecular genetics that most hybrids are F_1_, i.e., the direct offspring of *T. cristatus* and *T. marmoratus*, with low levels of subsequent backcrossing and bidirectional introgression (Vallée 1959; Arntzen and Wallis 1991; Arntzen et al. 2009; Arntzen 2024). The data analysis was, however, not geared up to the detection of triploid hybrids. Here, we deal with the questions if triploid hybrids exist in the *T. cristatus—T. marmoratus* system, how frequent they are, and how to distinguish them from F_1_ hybrids and hybrid backcrosses.

For KASP the results may be affected by the quality of the data. The KASP-results remains ‘uncalled’ by the associated software in the case of a low signal, but this also happens if the KASP coordinates fall in between homozygote and heterozygote clusters, as is to be expected for a triploid (Fig. 1). The technique of mMDS goes a long way in solving this issue, by separating biologically meaningful information that is projected on the first mMDS axis, from redundant information from low KASP signals that is projected onto the second mMDS axis. These issues apply primarily to KASP batch 1 that dealt with old material for which good quality DNA could not always be obtained (cf. Dasgupta and Sepulveda 2019).

The relatively high frequency (18%) of inferred triploids for KASP batch 1 may be biassed by the preferential sampling of suspected triploid and backcross hybrids. In batches 2 and 3 for which sampling was random with respect to hybrid phenotypes the number of triploids is 1 out of 26 hybrids (3.8%). From target capture data the frequency of triploids is one out of ten and from erythrocyte morphometrics it is one out of 38, altogether amounting to ca. 4% triploids amongst hybrids that themselves are a rare class (ca. 3%). *Triturus cristatus—T. marmoratus* F_1_ hybrid females with marmoratus type mtDNA are infrequent (2.5%), in line with a reciprocal asymmetry for mtDNA and sex (Arntzen et al. 2009, 2021). The hybrid individual identified as a triploid by gene capture belongs to this rare class of hybrids, underlining the peculiar status of this group.

Theoretically, triploids can arise in hybrids of diploid species in several ways. First, triploidy could result from the fusion of unreduced diploid gametes (usually oocytes) of one species with reduced haploid gametes of another, which seems the most likely in the *T. cristatus—T. marmoratus* system. If so, the different ratios of triploid types could be due to the fact that females (oocytes are more often diploid) of hybrids F_1_ (oocytes genotype CM) mate more often with males of *T. marmoratus* (M). Alternatively, it could result from the fusion of an oocyte of one species with two sperm of another species (polyspermy) or it could result from the induction of a triploid hybrid oocyte without fusion with the fertilizing sperm (as in gynogenetic salamanders of the genus *Ambystoma*; Bogart et al. 2009).

The one natural triploid *T. cristatus—T. marmoratus* F_1_ hybrid here illustrated is a unique finding (Fig. 5). It concerns a female with cristatus mtDNA. Its unusual hybrid phenotype, leaning towards *T. marmoratus*, was noted in the field, though at superficial inspection it might have passed as a an exceptionally large and sturdy but purebred *T. marmoratus*. The yellow ventral colouration derived from *T. cristatus* is less obvious than perhaps would be expected, but this may be due to the general trend observed in *T. cristatus*, in which the black belly spotting overtakes the orange base colour with age and body size (Arntzen and Teunis 1993; Hinneberg et al. 2020). The individual also stands out for a peculiar behaviour in annual pond attendance. Whilst it was observed in 2013, 2019, and 2023, it was *not* observed on another ten annual sampling sessions since it reached maturity around 2010. Chances that the individual was present in the pond but went unnoticed are slim, because the capture probability for *Triturus* newts at these events averaged at 97% (range 74% to 100%, excluding 2020 when no fieldwork was possible due to the COVID-19 pandemic) (Arntzen 2025a, Arntzen 2025b, JWA unpublished data). If such frequent skipping of annual breeding opportunities is typical for triploid hybrids, this would lead to their frequency being underestimated. Other peculiar, possibly transgressive, character states shown by this individual are longevity (17+ years), continued growth and large size (187 mm total length), but not body condition (Fig. 5). These issues are presumably interrelated, because resources not allocated to reproduction may be available for growth, stamina, and an increased survival probability (Cogălniceanu et al. 2020).

For the first time we show that triploidy infrequently occurs in a natural *Triturus* hybrid zone. Previously, triploidy was observed by karyotyping in three laboratory-produced *T. marmoratus* x *T. karelinii* backcrosses (Lantz and Callan 1954). Furthermore, flow cytometry in 226 field collected *T. dobrogicus* and 453 *T. cristatus* retrieved one triploid individual in either species (Litvinchuk and Borkin 2009). Cellular morphometrics does not distinguish between homozygotes and heterozygotes, so triploids identified with this technique may be autotriploids (e.g., ‘CCC’). It may be noted that autotriploids are readily obtained in the laboratory, under aberrant conditions such as increased hydraulic pressure or when lower temperatures are applied during the first cell division (Fischberg 1958; Ferrier and Jaylet 1978). However, because sampling was close to the *T. cristatus—T. dobrogicus* hybrid zone, a role for interspecies hybridization in triploid formation cannot be excluded.

Our recovery of triploid *Triturus* raises the question: Is triploidy of evolutionary importance in *Triturus* or, at another extreme, a dead-end street? At this stage of the research line, we are ambivalent to discuss the significance of triploids in the French *Triturus* hybrid zone, because we do not yet have reliable data on the reproductive capacity of the newly discovered triploid hybrids. However, in other amphibian groups such as species of the genus *Bufotes*, triploids may serve as a link in the formation of tetraploid species and participate in interploid gene flow (Betto-Colliard et al. 2018; Dufresnes et al. 2019; Litvinchuk et al. 2019). Triploidy in *Triturus* is particularly relevant to study in view of the chromosome-1 syndrome, which is a synapomorphy for the genus (Macgregor and Horner 1980). All adult *Triturus* possess two distinct versions of chromosome 1, 1A and 1B, that both miss a different chunk of chromosome that carries a large number of genes (France et al. 2025; De Visser et al. 2025b). Because chromosome 1 is inherited in a regular Mendelian fashion, 50% of offspring will possess 1A twice and lack 1B, or the other way around, and perish during embryonic development (Macgregor and Horner 1980; Sessions et al. 1988; Ridley 1993; Wielstra 2020). In theory, deviations from diploidy might provide an ‘escape route’ to the syndrome, because the production of 1A1B gametes would ensure that 100% of offspring would receive both 1A and 1B and, therefore, the full complement of genes required to survive. Evidently, such a work-around has not evolved ever since the basal split in *Triturus* at ca. 29 Ma (Stewart and Wiens 2025). This suggests that being a triploid itself comes with a strong fitness cost, presumably due to unbalanced dosage effects (France et al. 2025) and we conclude that triploidy in *Triturus* has no evolutionary future. With inferred triploid hybrids an estimated five times more numerous than backcrosses, the *T. cristatus—T. marmoratus* system finds itself positioned at what is truly the far end of the speciation continuum. If the documented low level of gene-flow beyond the early hybrid stages passes through diploids or triploids remains to be studied.

## Supplementary Material

Supplementary_information_123_esag006

## Data Availability

Data analyzed in this study are shown in Supplementary Information 1–3. Processing code for discerning less commonly observed allele frequencies in F_1_ hybrids is available at Zenodo https://zenodo.org/records/16938946.
